# Semantic Similarity to Known Second Language Words Impacts Learning of New Meanings

**DOI:** 10.3389/fpsyg.2018.02048

**Published:** 2018-10-24

**Authors:** Yuanyue Zhang, Baoguo Chen, Yixin Tang, Panpan Yao, Yao Lu

**Affiliations:** Beijing Key Laboratory of Applied Experimental Psychology, Faculty of Psychology, Beijing Normal University, Beijing, China

**Keywords:** second language learning, word learning, semantic similarity, ambiguous word, learning curve

## Abstract

Second language (L2) learners need to continually learn new L2 words as well as additional meanings of previously learned L2 words. The present study investigated the influence of semantic similarity on the growth curve of learning of artificially paired new meanings of previously known L2 words in Chinese–English bilinguals. The results of a translation recognition task showed that related meanings are learned faster and more accurately than unrelated meanings. The advantage of learning related new meaning persisted and increased for a week after learning the new meanings. These results suggest that semantic similarities impact the learning of new meanings for known L2 words, and that the shared features between previously known and new meanings of a word facilitate the process of incorporating the related new meaning into the lexical semantic network. Our results are discussed under the framework of the connectionist model.

## Introduction

Effective acquisition of vocabulary is essential to second language (L2) learners. Given the prevalence of ambiguous words in natural language, learning ambiguous L2 words is inevitable and vital.

One important characteristic of ambiguous word acquisition is that the new meanings, usually learned at different times from the originals, can be more related or less related semantically to the known meaning(s) of the words (Rodd et al., 2012), that is, their degree of relatedness is a continuous gradient. For example, the English noun *bank* has at least two semantically unrelated meanings: it can refer either to an organization where you can borrow and save money or the land along the side of a river. However, little is known about how semantic similarity influences the acquisition of new meanings of previously known L2 words. Exploring the learning of these new meanings provide a good opportunity to investigate how the previously known meaning impacts new meaning learning. For example, an already established semantic representation of a previously known meaning may facilitate or interfere with the process of bringing the new meaning into the lexical network. Thus, the present study aimed to investigate the influence of semantic similarity on learning of new meanings of previously learned L2 words.

### Previous Studies Relevant to Ambiguous L2 Word Acquisition

Previous studies have found that semantic similarity influences ambiguous L2 word acquisition (Bracken et al., 2017). In Bracken et al. (2017), English native speakers learned 43 novel German ambiguous words 3 times and then performed a translation recognition task immediately and 1 week later, in which they needed to judge whether L2–L1 word pairs were correctly paired. The results showed that the degree of semantic similarity between the two meanings of ambiguous L2 words was significantly correlated with learning performance in the 1-week-delayed translation recognition task (ACC: *r* = 0.34; RT: *r* = -0.52); that is, participants were faster and more accurate in the translation recognition task when the multiple meanings of one word were less related. Bracken et al. (2017) offered two possible accounts of the locus of semantic similarity. One is that new L2 forms are easier to map into two meanings if two meanings are interrelated. The other is that the competition between two unrelated meanings delays recognition, while more related meanings are not delayed in this way due to lack of competition between them during recognition (e.g., Rodd et al., 2002). The connectionist model characterizes word recognition as the process of retrieving a word’s meaning from an orthographic or phonologic representation to a stable semantic representation (see Rodd et al., 2004). Under this model, in language processing in general, many unrelated meanings are activated at the same time and compete with each other (e.g., Rodd et al., 2002; Armstrong and Plaut, 2008, 2016). To access a stable semantic representation, the connectionist model suggests, recurrent connections exist between semantic units and resolve the competition between activated unrelated meanings (Rodd et al., 2004; Armstrong and Plaut, 2008). Thus, performance may be worse when judging words with unrelated meanings than when judging words with related meanings in a lexical decision task.

However, L2 word acquisition includes multiple facets—not only learning novel word form and meaning but also learning additional meanings of already-learned words (Bogaards, 2001). A study using a sequential learning paradigm to investigate the learning of novel ambiguous L2 words found that the later-learned meaning representation is weaker than the first-learned one (Lu et al., 2017), which might be due to interference from the unrelated first-learned meaning; other studies have found that the activation of original meanings during the encoding of new meanings produces an interaction between new and existing word knowledge, a process that may facilitate the integration of new information (van Kesteren et al., 2012; Atir-Sharon et al., 2015; Schlichting et al., 2015). However, the studies mentioned above were focused on the learning of novel words (e.g., Atir-Sharon et al., 2015; Bracken et al., 2017; Lu et al., 2017), which is different from learning new meanings for familiar words. In the case of learning new ambiguous L2 words, neither the lexical formal representation nor the multiple semantic representation(s) have been well established during the learning process, meaning that this case cannot provide information about how the stable representation of previously known meanings influences the learning of new meanings. In the case of learning a new meaning of a familiar L2 word, the mapping from L2 form to old meaning is already established, and thus the old meaning may directly facilitate or interfere with the process of bringing the new meaning into the lexical network. Thus, exploring the learning of new meanings of previously known L2 words is beneficial to see how these new meanings are integrated into the lexical semantic network; however, this topic has rarely been investigated.

Indeed, to our knowledge, the study of Bogaards (2001) is the only previously published study investigating learning of new meanings of previously learned L2 words. In this study, Dutch speakers learned 3 types of items through French sentences and Dutch translations. Type 1 and Type 2 were new meanings of already acquired words. Type 1 were new meanings related to the acquired words; for example, learners learned the related new meaning “(a) drawing” of the word *crayon*, which is known to mean “pencil.” Type 2 were new meanings unrelated to already acquired words. For example, learners learned the unrelated new meaning “money” of the word *blé*, which is known to mean “wheat.” Type 3 were totally new words which had similar meanings to the meanings to be learned in Types 1 and 2. For example, learners learned the new word *fusain*, which has a meaning of “drawing,” similar to the to-be-learned meaning of *crayon*; or *flouse*, which has a meaning of “money,” similar to the to-be-learned meaning of *blé*. There were 7 Type 1 and 7 Type 2 items; Type 3, as a control condition, included 14 words. A 4-to-1 forced-choice test, which required learners to choose the right French translation equivalent of a given Dutch word, was administered immediately after learning, and a surprising word production task, which required learners to translate French words into Dutch, was implemented 3 weeks later. The results of both the immediate 4-to-1 forced-choice test and the 3-week-delayed word production test showed no difference in accuracy between learning of related and unrelated new meanings; however, the general accuracy of the new meanings (Types 1, 2) was higher than that of the new words (Type 3) in the delayed production test. In sum, the study suggested that semantic similarity has no impact on learning the new meanings of already known words and that it is harder to learn and recall novel words than it is to learn and recall new meanings of already known words.

Bogaards (2001) did not find a semantic similarity effect in learning of new meaning of previously known L2 words, which might, however, have been due to limited or narrow learning materials and tasks. In Bogaards (2001), participants needed to learn 7 words of each of the types described above. In the immediate forced-choice task, they successfully learned 6.3 (±0.94) words for related new meanings and 5.8 (±1.29) words for unrelated new meanings. After 3 weeks, participants went back to the lab and performed a surprising L2–L1 production task. In the task, participants retained very few items: 1.21 (±0.93) words for related new meanings, 1.13 (±1.00) words for unrelated new meanings. Therefore, one possible explanation of the non-significant semantic similarity effect is that the limited learning materials and the harder L2–L1 production test led to a ceiling effect in accuracy on the immediate task and a floor effect on the delayed task. If so, as a consequence, it would have been hard for Bogaards to clearly determine the impact of semantic similarity.

According to the connectionist model, semantic similarity might influence the learning of late-learned meanings of previously known words. That is, the connectionist model suggests that a semantic representation consists of multiple shareable semantic features (e.g., Rodd et al., 2004; Armstrong and Plaut, 2008), and that learning new meanings entails strengthening the connection between these multiple semantic features. Accordingly, learning a new word means integrating it into the existing lexical semantic network, as defined by these features; the more similar the new and the previously known meanings, the more semantic features they share. If so, it should be easier to learn a related new meaning of a previously known word than an unrelated meaning. To test this possibility, it is necessary to further explore semantic similarity effects on learning the new meanings of previously learned L2 words.

In addition, Bogaards (2001) did not track change in learning performance after every learning task (e.g., learning through L2 sentences and L1 translation equivalents). Therefore, the results could not reveal how semantic similarity influences the time course of learning. So, the second goal of the present research was to examine whether semantic similarity has an impact on the growth curve of learning new meanings. Specifically, our interest is in the rate and the shape of change of learning each type of new meaning of previously known words.

### The Current Study

In sum, the present study aimed to explore the influence of semantic similarity on learning new meanings of previously learned L2 words. Specifically, does semantic similarity impact the growth curve of learning these meanings?

In the current study, a new meaning (given in the L1) was paired to a familiar L2 word, at one of 2 levels: related to the old meaning [e.g., “forest”-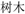 *shumu* “trees”] or unrelated [e.g., “student”-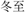 *dongzhi* “midwinter”]. Participants learned this new meaning repeatedly, 9 times over 3 consecutive days (a length of time informed by previous studies in lab-based L2 vocabulary acquisition (Liu et al., 2007; Lu et al., 2017). In Lu et al. (2017), 30 novel English words (15 unambiguous, 15 ambiguous) were successfully learned by native-Chinese-speaking English-learners over 4 consecutive days (learners reached overall accuracy of 90% in a cross-language semantic relatedness judgment task, which required learners to judge whether the English word they learned was semantically related to a Chinese word), while Liu et al. (2007) successfully taught 60 L2 words in 3–4 days, with a total of 6–7 h of training (accuracy on both the naming task and the category judgment task was above 90%. In the naming task, learners were required to read the L2 words aloud; in the category judgment task, learners were required to judge the semantic category membership of L2 words: animal, man-made object, or neutral object). Note, however, that learners in these previous studies needed to learn both the new meaning and the new form of the L2 words, which is more difficult than learning the new meanings only (Bogaards, 2001), such as in the current study. Thus, we maintained the 3 days of consecutive learning with a total of 10–11 h of training but taught 240 new meanings in the current study. Our pilot study found that 3 days of learning was appropriate to tracking the learning curve—not too long, risking boring the learners, nor too short, and hence leaving learners unable to reach stable learning performance. We set 240 new meanings with the aim of maintaining appropriate difficulty of the learning task to observe the changing performance.

In order to find out whether there was a semantic similarity effect on the learning, each learning cycle was followed by an immediate test to measure memory of the new meanings. The test consisted of a translation recognition task in which participants were required to decide whether L2 and L1 words were correctly paired (Degani and Tokowicz, 2010; de Groot, 2011; Degani et al., 2014; Pu et al., 2016). We used the translation recognition task as our main test task because it is less difficult than production tasks or cue recall tasks (Laufer et al., 2004; de Groot, 2011). We can draw the growth curve by continuing to measure learning performance: the slopes reflect the change rate after each time learning the new meaning (hereafter, the learning speed). By comparing the learning speed of related and unrelated new meaning conditions, we intended to find out whether semantic similarity impacts learning: if so, the learning speeds of the two new meaning conditions would be different, and if not, the speed would be the same.

Following the connectionist model, we predicted that semantic similarity would indeed influence the learning of new meaning, and the speed of learning of related new meanings would be faster than that of unrelated new meanings.

## Materials and Methods

### Participants

Thirty-three Chinese native speakers (17 females; age: 21.41 ± 3.36 years) with low to intermediate proficiency in English were recruited from several universities in Beijing. All participants reported having normal or corrected-to-normal vision. Data from a total of 4 (male) participants were excluded because they dropped out of the experiment; thus, analyses were conducted on the final set of 29 participants. Prior to data collection, ethical approval was obtained from the Committee of Protection of Subjects at Beijing Normal University. All the participants signed the written informed consent form and received a small, variable payment for their participation. In order to motivate their learning, participants were told that the amount of the payment would be determined according to their learning performance.

The Oxford Placement Test (OPT) and self-assessment ratings were used to assess the English proficiency of the participants. The OPT is made up of 25 multiple-choice questions and a cloze test, with a total score of 50. Self-assessment is on a scale ranging from 1 (very poor) to 6 (excellent) for English listening, speaking, reading, and writing skills. The participants in the present study were late unbalanced Chinese–English bilinguals (AoA: 8.79 ± 2.26, duration of learning English: 12.62 ± 3.36), whose English proficiency was moderate: average 3.48 ± 0.96) for listening, 3.36 ± 0.99 for speaking, 3.28 ± 1.17 for reading, and 3.52 ± 1.16 for writing. Average OPT score was 37.83 ± 4.5.

### Design

This study used a session within-subjects design. Word type/similarity values and testing blocks were used as predictors of reaction time and accuracy.

### Stimuli

#### Learning Stimulus

Participants were required to learn 360 English–Chinese word pairs in which the English words were familiar and highly recognizable and have one single dominant translation, taken from the Wen and van Heuven (2017) translation norms. They were requested to learn the paired Chinese 2-character word meaning for each word. One-third of the English words were paired with new Chinese 2-character words semantically related to the word’s existing meaning [e.g., “forest”-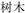 (*shumu*) “trees”; the related new meaning condition], another one-third were paired with semantically unrelated Chinese words [e.g., “student”-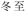 (*dongzhi*) “midwinter”; the unrelated new meaning condition], and the final one-third were paired with Chinese translation equivalents [e.g., “audience”-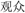 (*guanzhong*) “audience”; the unambiguous words condition].

All the Chinese words were chosen from the Beijing Language and Culture University (1986). The criterion for L1 words chosen as related to L2 words was fitting at least one of the following: (1) the 2 words are synonyms or near-synonyms; (2) one word is a hyponym of the other word; (3) the 2 words are co-hyponyms of the same hypernym; or (4) the 2 words share some conceptual feature. Lexical properties of L2 and L1 are matched across 3 types of condition (related new meanings, unrelated new meanings, and unambiguous words conditions; see Table 1).

**Table 1:** Lexical properties of three types of English words and their paired Chinese meanings, Mean (SD).

	Lexical properties	Related	Unrelated	Unambiguous
English word	Familiarity	6.60(0.49)	6.57(0.45)	6.55(0.44)
	Word length	6.43(2.18)	6.42(2.10)	6.19(2.03)
	Word frequency	76.86(83.22)	80.70(183.22)	82.24(123.66)
	NON	3.33(4.78)	3.36(5.24)	3.50(5.13)
	Semantic similarity	5.64(0.54)	1.78(0.55)	
Chinese word	Word frequency	34.52(43.16)	36.08(42.03)	56.97(166.44)


*The lexical properties of L2 words (English words) are from the MCWord database (Medler and Binder, 2005). NON, number of orthographic neighborhood. The Lexical properties of L1 words (Chinese words) are from the Beijing Language and Culture University (1986).*

No significant differences were observed for the following lexical properties among the 3 types of condition: word length of English words, *F* (2,357) = 0.49, *p* = 0.61, η^2^ = 0.003; frequency (per millions), *F*(2,357) = 0.05, *p* = 0.95, η^2^ = 0.000; orthographic neighborhood statistic for English words, *F*(2,357) = 0.04, *p* = 0.96, η^2^ = 0.000; word frequency of Chinese words (per millions), *F*(2,357) = 1.81, *p* = 0.17, η^2^ = 0.010. In order to help participants focusing on learning L2-L1 word pairs in each training cycle, 40 English pseudowords paired with Chinese 2-character words were included as probes for the learning task [e.g., “meabon”-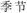 (*jijie*) “season”]. Participants were asked to press the “J” key on the keyboard when they encountered a pseudoword. Since the proportion of L2 pseudoword–L1 word pairs out of the total word pairs was small (10%), participants needed to focus on every learning trial in order to detect these small-probability events (see also Pu et al., 2016).

As many researchers have suggested that semantic similarity is a continuous variable, from very unrelated at one end to very related at the other end (Klein and Murphy, 2001; Klepousniotou, 2002; Klepousniotou and Baum, 2007; Klepousniotou et al., 2008, 2012; Jager et al., 2016), semantic similarity between all new meanings and existing meanings of L2 words was also independently established through a rating study. Nineteen native Chinese speakers who did not participate the learning task were asked to rate the familiarity of the English words (1: very unfamiliar, 7: very familiar), as well as the semantic similarity between the existing meaning and the new meanings of each English word, on a scale ranging from 1 (very unrelated) to 7 (very related). The scores are given in Table 1. A one-way ANOVA was used to test if there was any difference in familiarity among the 3 types of words; no significant difference was observed in the familiarity of L2 items, *F*(2,36) = 2.27, *p* = 0.12, η^2^ = 0.112. Another one-way ANOVA was used to test if there was any difference in semantic similarity for 2 types of words (related words, unrelated words); the semantic similarity values for the related new meaning condition (Mean = 5.64, *SD* = 0.54) were significant higher than those for the unrelated new meaning condition (Mean = 1.78, SD = 0.55), *F*(1,18) = 1012.84, *p* < 0.001, η^2^ = 0.98. The semantic similarity value of each word pair was used as the predictor in the following data analysis.

#### Testing Stimulus

In the pre-learning session, a translation recognition task was adopted to test if participants were familiar with the L2 words that we selected. Three hundred and sixty English words were included, half of them paired with their Chinese translation equivalents and the other half randomly paired with other Chinese translation words. Participants were asked to press the key to indicate whether L2–L1 words are correctly or wrongly paired; in order to make sure no more than 3 sequential trials were of the same type, the order of the 360 English–Chinese word pairs was pseudorandomized.

In the learning session and the post-learning session, the translation recognition task was adopted to test learning performance. As before, 360 L2 words and their learned meanings were included, half of them paired with correct learned meanings and the other half randomly paired with other meanings. In order to reduce the effect of repeated testing on learning, each testing block only included 90 L2–L1 word pairs (half correctly paired, half wrongly paired), that is, a quarter of the total pairs. We created 5 word-pair lists, with different presentation orders. Three lists were used in the 3-day learning session and the other 2 lists in the 2 post-learning tests. Word pairs in the lists were pseudo-randomly sorted. Two versions of the lists were created for counterbalancing across participants.

#### Learning and Test Procedure

**FIGURE 1:**
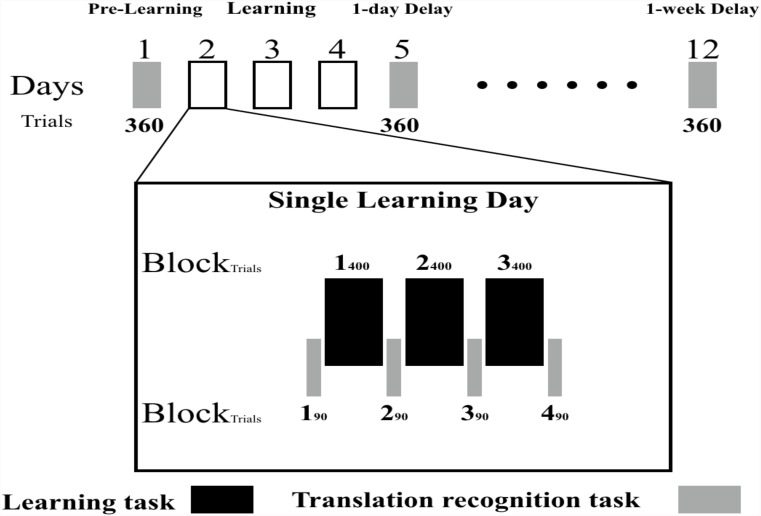
The learning and test procedure: the black rectangle in the picture represents the learning task; the gray rectangle in the picture represent the testing task; the black box represents the learning session. Participants took part in 1 pre-learning, 3 days of learning and 2 post-learning sessions. During the learning session, participants performed 4 blocks of translation recognition task (90 trials for each block) and 3 cycles of learning (400 trials for each cycle) in turn. During the presentation of word pairs, if the English word was a real word, participants were required to memorize the word pair; if the English word was a pseudoword, participants were required to press the “J” key to go to the next trial. A translation recognition task was used 1 day before learning session for the purpose of investigating the familiarity to those L2 words. The translation recognition task in post-learning session was used to test the learning effect of new meaning.

The procedure was conducted over 2 weeks. The first day served as the pre-learning session, and the next consecutive 3 days served as the learning session. On each day, participants completed the learning task (3 blocks) and the translation recognition task (4 blocks). The fifth day and the 12th day served as the post-learning sessions, in each of which participants were asked to complete a translation recognition task. The learning and testing procedure are presented in Figure 1.

The learning section lasted for 3 days. Each day, participants were required to perform a learning task (3 blocks) and a translation recognition task (4 blocks, with one at the beginning of the learning task and one after each block of learning task, see Figure 1). The translation recognition task and the learning task were performed alternately. Each learning day started with a block of translation recognition task, then a block of learning task, then a block of translation recognition task, and so on. Thus, there were 12 blocks of translation recognition task and 9 blocks of learning task over the whole learning section. Each block of the learning task was one training cycle (400 trials, 360 critical L2–L1 pairs, 40 pseudoword-L1 pairs). In order to reduce the effect of repeated testing on learning, each block of the test only included a quarter of critical learning stimuli (90 trials).

In the learning task, each L2–L1 word pair was shown for 3000 ms after a fixation cross shown for 800 ms. During the presentation of word pairs, if the English word was a real word, participants were required to memorize the word pair; if the English word was a pseudoword, participants were required to press the “J” key to go to the next trial. This additional go/nogo lexical decision task helped to make sure that participants were focusing on the word pairs.

In the translation recognition task, each trial started with a fixation cross presented for 800 ms in the center of the screen. Then, the L2–L1 word pair appeared on the screen until a response was made or until 3000 ms elapsed with no response. Participants were asked to judge whether L2 and L1 words were correctly paired; “yes” or “no” responses were respectively made by pressing the “F” or “J” on the keyboard. The keys for yes/no were counterbalanced between participants. In the beginning of each block, there were 12 practice trials. At the end of each block, the accuracy for that current block was shown; participants could adjust their learning according to the feedback. For example, participants could keep learning if their accuracy was too low.

## Results

### Pre-learning Session

Accuracy on the translation recognition task was greater than 85% for all participants. The Friedman test was used to find any difference in accuracy among the 3 types of word, and found no significant difference o (χ^2^ = 2.062, *df* = 2, *p* = 0.357), meaning all the participants were familiar with the L2 words and showed no bias on any type of L2 word.

### Learning Session

#### Reaction Time

**FIGURE 2:**
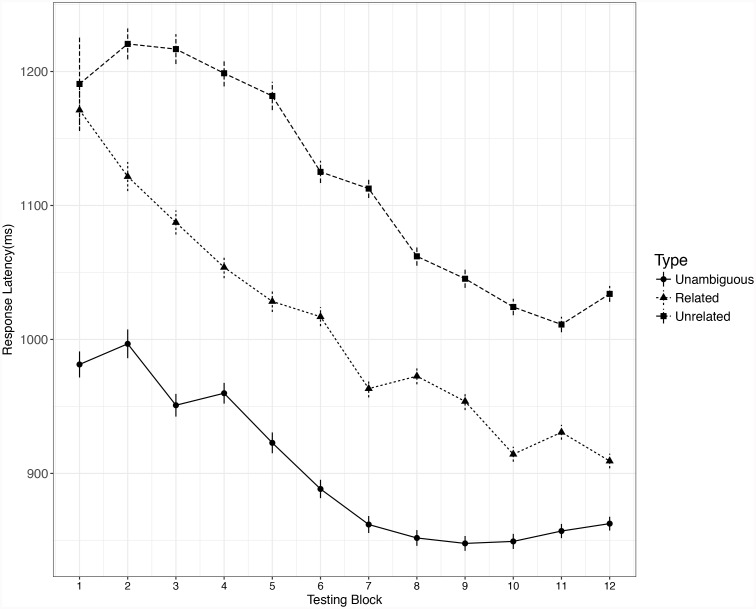
Growth curve of RT data for three different types of words. Error bar represents ± SE.

**FIGURE 3:**
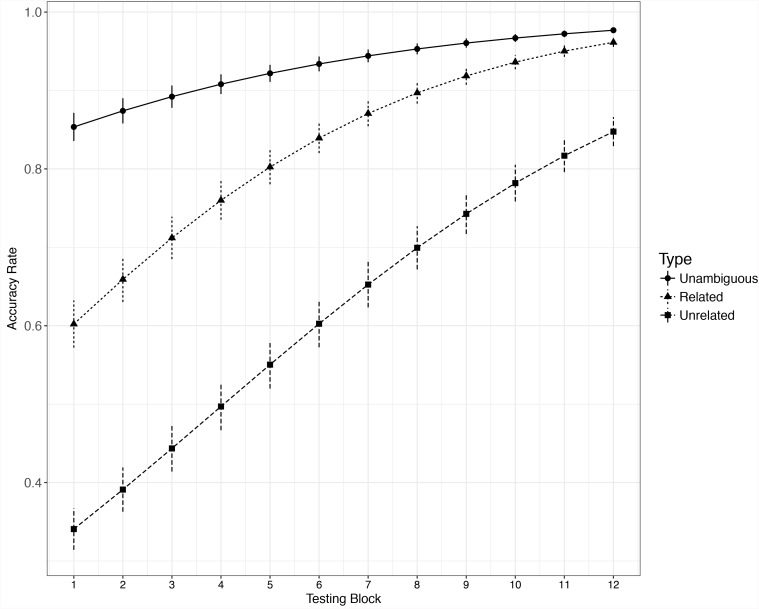
Growth curve of accuracy data for three different types of words. Error bar represents ± SE.

Only the RT data for correctly responded yes trials were analyzed. For each participant, the extreme outliers (RT < 200 ms or RT > 2500 ms) and response latencies beyond Mean ± 3 SD were excluded (2.4%) from the dataset (see, e.g., Jager et al., 2016; Lu et al., 2017). Changes in vocabulary learning across time are often non-linear (Bisson et al., 2014; Murre, 2014), so we conducted growth curve analyses including higher-order polynomial terms (Baayen et al., 2008; Cunnings, 2012; Mirman, 2014), adding variable and higher-order polynomial terms step by step until the model could not converge; then the controlled variables that reached significance and improved model fit significantly (word length, word frequency, number of orthographic neighborhoods) were added. Both RT and accuracy data were analyzed using the lme4 package tool (Bates et al., 2014) in the R computing environment (R Core Team, 2013). The learning curve of three types of words were presented in Figure 2 (the Reaction Time data) and Figure 3 (the accuracy data).

The final model was built with word types, block^1^, block^2^, block^3^, word length (which was the only significant control variable), and the interaction between word type and all the block terms (block^1^, block^2^, block^3^) as fixed effects, with random by-participant and by-item slopes for the block. Within word type, reverse Helmert coding was used to define 2 planned contrasts: (a) related new meaning = -1/2 versus unrelated new meaning = 1/2, and (b) unambiguous word = 2/3 versus related or unrelated new meaning = -1/3. Likelihood ratio tests were used to evaluate the main effects and interactions, and the significance of individual model coefficients was obtained using the *t* statistic in the model summary. The main effect of word type was significant, χ^2^ = 52.54, *df* = 2, *p* < 0.001. The coefficient for the first contrast of word type (related vs. unrelated new meaning) was not significant, *b* = 50.21, *t* = 1.47, *p* = 0.139, which means no significant difference was found in the reaction times for recognizing related versus unrelated new meanings before learning. The coefficient for the second contrast of word type (unambiguous word vs. the 2 types of new meanings of words) was significant, *b* = -175.30, *t* = -7.49, *p* < 0.001. A Bonferroni-corrected Tukey test was conducted using the “glht” function from the “multcomp” package (Hothorn et al., 2008) for R. The results for these tests were consistent with those from the model summary: both the related (*z* = -6.01, *p* < 0.001) and unrelated (*z* = -6.19, *p* < 0.001) new meaning conditions were responded to significantly more slowly than the unambiguous word condition.

A likelihood ratio test for the full model and models without the interaction term showed that the critical word type × each block terms (block^1^, block^2^, block^3^) interactions were significant (block^1^: χ^2^ = 8.12, *df* = 2, *p* = 0.017; block^2^: χ^2^ = 8.40, *df* = 2, *p* = 0.015; block^3^: χ^2^ = 7.43, *df* = 2, *p* = 0.024). The significance tests for model coefficients showed that the first contrast of word type (related vs. unrelated new meaning) significantly interacted with all the block terms (block^1^: *b* = 67.38, *t* = 2.85, *p* = 0.004; block^2^: *b* = -13.44, *t* = -2.87, *p* = 0.004; block^3^: *b* = 0.72, *t* = 2.72, *p* = 0.007), which suggests that the slope of the related new meaning condition is steeper than that of the unrelated new meaning condition. In other words, learning related new meanings is faster than learning unrelated new meanings. However, the second contrast of word type (unambiguous words vs. 2 types of new meaning) did not interact with any block terms (block^1^: *b* = -13.03, *t* = -0.77, *p* = 0.439; block^2^: *b* = 3.28, *t* = 0.95, *p* = 0.344; block^3^: *b* = -0.13, *t* = -0.65, *p* = 0.514).

To determine whether there was a significant difference between the slope of unambiguous words and each of the 2 types of new meaning, we compared the response latency for each type of new meaning to that in the unambiguous word condition (i.e., related new meaning vs. unambiguous words, unrelated new meaning vs. unambiguous words). For each subset analysis, a full model was established with effect-coded word type (one type of new meaning = 1, unambiguous words = 0), block^1^, block^2^, block^3^, and word length as fixed factors, and with by-participant and by-item random intercepts and slopes for the block. The full model did not converge for either the related and unrelated word type subsets, so the by-item intercept was removed, as it accounted for the least variance. The model coefficient significance tests showed that there were significantly steeper slopes for both related new meanings (block^1^: *b* = -36.09, *t* = -2.58, *p* = 0.009; block^2^: *b* = 6.44, *t* = 2.23, *p* = 0.026; block^3^: *b* = -0.39, *t* = -2.33, *p* = 0.019) and unrelated new meanings (block^1^: *b* = 41.46, *t* = 2.094, *p* = 0.036; block^2^: *b* = -9.2, *t* = -2.365, *p* = 0.018; block^3^: *b* = 0.46, *t* = 2.103, *p* = 0.036) relative to unambiguous words.

To find out whether there was an influence of semantic similarity on learning the new meanings of previously known words, and if so, how the impact of semantic similarity changed across blocks, we first calculated the correlation coefficient value between semantic similarity value and response latency in each testing block (hereafter: *r_i_*; *i* for the block: 1–12). Semantic similarity was negatively correlated with response latency in each block except the first block, which is similar to the finding of Bracken et al. (2017). Before learning, the semantic similarity value was positively correlated with response latency (*r_1_* = 0.3, *p* = 0.04) in the first block, which suggests that the time it took to recognize a new paired L1 word increased with the similarity between the new and previously known meanings. Second, we calculated the Bonferroni-corrected Pearson correlation coefficient (*r_block_*) between *r_i_* (*i* > 1) and testing block (2–12) in order to find any change tendency in the correlation between semantic similarity and response latency (*r_i_*, calculated above) after learning. We reasoned that if the semantic similarity effect enlarged after every round of learning, the testing block (2–12) would be negatively correlated with *r_i_* (*i* > 1), while if the semantic similarity effect decreased, the testing block (2–12) would be positively correlated with *r_i_* (*i* > 1). The result showed that testing block positively correlated with *r_i_* (the correlation between semantic similarity and response latency), *r_block_* = 0.328, *p* < 0.001. This result suggests that the influence of semantic similarity gradually decreased across blocks.

#### Accuracy

As with the RT data, a full model was built to look into the growth curve of the learning of the 3 types of words, with block and reverse-Helmert-coded word type as fixed effects and with by-participant random intercepts. The main effect of word type was significant, χ^2^ = 787.55, *df* = 2, *p* < 0.001, as was the coefficient for the first contrast of word type (related vs. unrelated new meaning condition), *b* = -1.19, *z* = -14.21, *p* < 0.001. The coefficient for the second contrast of word type (unambiguous words vs. 2 types of new meaning) was also significant, *b* = 2.08, *z* = 21.33, *p* < 0.001. Tukey tests with Bonferroni-adjusted results were consistent with those from the model summary, recognizing both the related (*z* = 13.79, *p* < 0.001) and unrelated (*z* = 25.50, *p* < 0.001) new meaning conditions as less accurate than the unambiguous words. Model comparison results showed that the word type × block interaction was significant (χ^2^ = 15.57, *df* = 2, *p* < 0.001). The significance tests for model coefficients showed that block significantly interacted with both the first contrast of word type (related vs. unrelated new meaning, *b* = -0.03, *z* = -2.16, *p* = 0.03) and the second contrast (unambiguous word vs. 2 types of new meaning, *b* = -0.06, *z* = -3.57, *p* < 0.001), which suggests that the slope of the related new meaning condition is steeper than that of the unrelated new meaning condition.

In order to explore the difference in learning speed between unambiguous words and each type of new meaning, we compared accuracy in each new meaning condition to that in the unambiguous words condition (i.e., related new meanings vs. unambiguous words, unrelated new meanings vs. unambiguous words, respectively). For each subset analysis, a full model was built, with word type (one type of new meaning or unambiguous word), block, and interaction of block and word type as fixed factors, and with a by-participant random intercept. The model coefficient significance test result showed that the slopes of both related new meanings (*b* = 0.08, *z* = 3.85, *p* < 0.001) and unrelated new meanings (*b* = 0.05, *z* = 2.59, *p* = 0.009) were steeper than that of unambiguous words.

In summary, both RT and accuracy data showed a slope for the related new meaning condition that was significantly steeper than that for the unrelated new meaning condition. The slope of the growth curve reflects learning speed; thus, our results showed that learning was faster for related new meanings than for unrelated new meanings. Also, the slope of the new meanings condition was steeper than that of the unambiguous words condition, which reflects that the speed of learning new meanings was faster than the change rate in recognition of familiar unambiguous L2 words.

### Post-learning Session

A full model was fitted to the RT data to explore the retention of effects of learning, with word type reversed-Helmert-coded, testing session coded as treatment (1-day-delayed = 0, 1-week-delayed = 1), word length and the interaction of word type and testing session as fixed effects, and random by-participant and by-item slopes for testing session and L2 word length. The high accuracy of related new meanings and unambiguous words, which was near the ceiling, could undermine the accuracy of logistic regression; thus, the Friedman test was used to test the accuracy data for both 1-day-delayed and 1-week-delayed testing. Only the accuracy data for the yes trials were included in this analysis.

**FIGURE 4:**
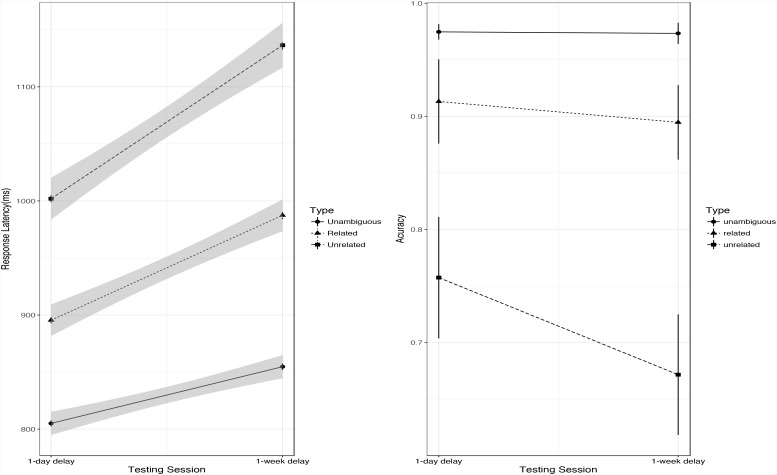
Accuracy **(right)** and RT **(left)** data for three different types of words in post-learning session, Error bar represents ± SE.

The RT data and the accuracy data for the 2 post-learning sessions are shown in Table 2. In the full model, participants reacted faster in the 1-day-delayed test than the 1-week-delayed test (*b* = 91.79, *t* = 8.413, *p* < 0.001). In the 2 delayed tests, recognizing related new meanings was faster than recognizing unrelated new meanings (1-day-delayed test: *b* = 109.32, *t* = 9.12, *p* < 0.001; 1-week-delayed test: *b* = 154.08, *t* = 11.12, *p* < 0.001). The second type of contrast (unambiguous words vs. 2 new meaning conditions: related and unrelated) was significant in both tests (1-day-delayed test: *b* = -138.68, *t* = -13.78, *p* < 0.001; 1-week-delayed test: *b* = -205.04, *t* = -17.81, *p* < 0.001). Tukey tests with Bonferroni-adjusted results showed that recognizing unambiguous L2 words was faster than recognizing related new meanings (1-day-delayed test: *b* = -84.02, *z* = -7.32, *p* < 0.001; 1-week-delayed test: *b* = -128.01, *z* = -9.768, *p* < 0.001) or unrelated new meanings (1-day-delayed test: *b* = -193.35, *z* = -16.21, *p* < 0.001; 1-week-delayed test: *b* = -282.08, *z* = -20.502, *p* < 0.001). The interaction between testing session and word type was significant (χ^2^ = 32.45, *df* = 2, *p* < 0.001); specifically, the extent of decrease for unrelated new meanings between the 1-day-delayed test and the 1-week-delayed test was significantly larger than that for related new meanings, *b* = 44.756, *t* = 2.911, *p* = 0.004. The results of subset analysis with each new meaning condition and the unambiguous condition showed that the decreases in the new meaning conditions (related new meaning: *b* = 43.359, *t* = 3.23, *p* = 0.001; unrelated new meaning: *b* = 87.50, *t* = 6.01, *p* < 0.001) between the 2 tests were significantly larger than for unambiguous words (see the left panel in Figure 4).

**Table 2:** Mean (SD) of RT (ms) and accuracy data (%) for three types of words in the post-learning session.

Word type	Response time		Accuracy	
	
	1-day-delayed	1-week-delayed	1-day-delayed	1-week-delayed
Unambiguous word	805 (193.20)	854 (227.97)	97 (0.05)	96 (0.06)
Related word	895 (244.46)	987 (306.89)	93 (0.11)	92 (0.10)
Unrelated word	1001 (323.22)	1136 (352.24)	85 (0.16)	80 (0.15)


A Shapiro–Wilk test confirmed that participants’ performance across the post-learning session was not normally distributed [*W*(173) = 0.734, *p* < 0.001]. Friedman test results suggested that the accuracy of the 3 types of words was significantly different on both the 1-day-delayed test (χ^2^ = 39.755, *df* = 2, *p* < 0.001) and the 1-week-delayed test (χ^2^ = 45.243, *df* = 2, *p* < 0.001); in the 1-day-delayed test, the accuracy of recognizing related new meanings of L2 words and that of recognizing unambiguous words were higher than that of recognizing unrelated new meanings of L2 words (related new meanings: *W* = 213, *p* = 0.004; unambiguous words: *W* = 132, *p* < 0.001), while no significant difference was found between related words and unambiguous words (*W* = 329, *p* = 0.456). In the 1-week-delayed test, the accuracy of recognizing L2 unambiguous words was higher than that of recognizing L2 words paired with related new meanings (*W* = 245.5, *p* = 0.02), which was in turn higher than that for L2 words paired with unrelated new meanings (*W* = 182.5, *p* < 0.001; see the right panel in Figure 4). These results suggest that the semantic similarity effect had become larger after a week.

Both the RT data and the accuracy data thus show that learning performance on unrelated new meanings was worse than that on related new meanings, which was in turn worse than that on familiar unambiguous words, and that the differences in the learning of these 3 types of words became bigger after a week.

## Discussion

The present study aimed to explore the extent to which semantic similarity impacts the learning of new meanings of familiar L2 words, specifically its influence on the growth curve(s) of learning new meanings of familiar L2 words. Consistent with our hypothesis, the growth curve of learning related meanings was significantly different from that for unrelated meanings, as indicated by their respective slopes: the slope for related meanings was steeper than that for unrelated meanings, meaning that the speed of learning related meanings was faster than that of learning unrelated meanings. These semantic similarity effects, reflected by the slopes of the growth curves, support the view that semantic similarity impacts the learning of new meanings of familiar L2 words: learning related new meanings is faster and easier than learning unrelated new meaning. This impact of semantic similarity became bigger even 1 week later. In this section, the results will be explained in the light of the connectionist model.

The connectionist model (e.g., Rodd et al., 2004; Armstrong and Plaut, 2008) considers the learning of a new meaning to involve integrating it into the existing semantic network through semantic features shared between new and old meanings: the more similar the old and new meaning, the more semantic features shared. Thus, semantic similarity should impact the growing speed of learning new meanings. The connectionist model explains our results well: shared features between the known and related new meanings make it easier for learners to integrate related new meanings into their existing semantic network of L2 words, while low learning speed of unrelated new meanings may be attributed to difficulties integrating these meanings and constructing the right L2-form-to-new-meaning mapping relationship. One possible explanation of why integrating new unrelated meanings is difficult is that the non-shared features of the old meaning may interfere with the integration of the new meaning, and that to overcome this interference, inhibitory control takes part in the learning of new unrelated meanings. This is in line with our previous research, in which inhibitory control ability positively predicted learning performance on unrelated new meanings (Lu et al., 2017). Therefore, the current study lends further credit to the connectionist model.

In the post-learning session, the semantic similarity effect had increased by 1 week later, reflecting a large-scale decrease in recognition of the unrelated new meanings. These results suggest that the semantic representation of unrelated new meanings is weaker than that of related new meanings, and that therefore the unrelated meanings are more vulnerable to retreat than the related meanings. This persistent, enlarged semantic similarity effect lends additional credence to the view that difficulty integrating unrelated new meanings results in difficulty processing ambiguous words with multiple unrelated meanings (Rodd et al., 2012).

Our results are similar to the findings in Rodd et al. (2012), which found that semantic relatedness impacts new meaning learning for L1 familiar words. In this study, native-English-speaking participants were required to learn new meanings of previously known English words from short passages. After this learning task, participants recalled the properties of the new meaning (the cued-recall test) and decided whether a given English word was a real word or not (the lexical decision task). The results showed that adult learners correctly recognized words faster when the new meaning was semantically related to the previously known meaning than when it was a new, unrelated meaning. Also, participants recalled more properties of related new meanings than of unrelated new meanings, in both the immediate and the delayed cued-recall test. Together with Rodd et al. (2012), our results suggest that the influence of semantic similarity in learning the new meanings of old words may be similar across L1 and L2.

Learning a new meaning of a familiar word is different from learning a new word (Bogaards, 2001). In the former case, the L2 form and the mapping from L2 form to old meaning are already established, and thus the old meaning may directly facilitate the process of bringing in the new, related meaning (van Kesteren et al., 2012; Schlichting et al., 2015), or, conversely, interfere with establishing the new mapping from L2 to new, unrelated meaning. In our study, we found that the semantic similarity effect arises in the learning process. Bracken et al. (2017), in contrast, found that semantic similarity effects only arose 1 week later, rather than immediately after the learning session. It is possible that this difference may be caused by the different types of learning in the current study and in Bracken et al. (2017); participants in Bracken et al. (2017) were required to learn brand-new ambiguous L2 words, that is, to create a new L2 form and map this new L2 form into two meanings, whereas in our study, the learners were familiar with the L2 forms, and the mappings from the L2 forms to the meanings were already established.

Bracken et al. (2017) offered two possible loci for the semantic similarity effect: (1) the establishment of form-meaning mapping or (2) the retrieval process, after form-meaning mapping was well established. In the current study, we tracked change in learning performance after every learning cycle and used the slopes of the growth curves to reflect learning speed. By depicting the growth curve and comparing growing speed for different types of word, our research provides supporting evidence that the semantic similarity effect might arise in the process of establishment of form-meaning mapping. Note, however, that learning is a long-term and evolving process, and we cannot roughly separate the learning and retrieving processes based on whether a learning task is being performed or not. The different speeds of learning related and unrelated new meanings and the gradually fading correlation between semantic similarity value and testing block that we found can only serve as indirect evidence to support the view that a semantic similarity effect might arise from the process of establishing form-meaning mapping.

Two possible explanations might lead to interpretations of the current result: the familiarity effect and decision strategy. In the first block of testing, we found a significant difference in accuracy between related and unrelated new meanings, leading to the possible explanation that the semantic similarity effect we found in learning may be a familiarity effect, in which learners are more familiar with the related meaning condition than the unrelated meaning condition. Note, however, that no significant difference was found in the RT data between related and unrelated new meanings before learning. As a previous study has suggested that familiarity improves word recognition (Gernsbacher, 1984), it would seem weird that no difference was found between the conditions if participants were more familiar with related than unrelated new meanings. Moreover, the Pearson correlation test found that semantic similarity was positively correlated with response latency in the first block of testing, which suggests that the more new and old meanings shared similarities, the more time was spent on recognizing whether L1 words conveyed the right meanings for L2 words. Thus, we speculate that the significant difference we found in accuracy data for the first block of testing might because learners were uncertain whether a semantically related L1 word provided the “right” meaning of the L2 word, rather than because of familiarity. Specifically, relative to the unrelated new meaning condition, the significantly high accuracy of the related new meaning condition in the first block of testing might reflects that the learners were more inclined to treat a related new meaning than an unrelated new meaning one as a right new meaning before learning.

Another possible explanation of the difference is related to decision strategy in the translation recognition task, such as deciding based on word-pairs’ semantic similarity. We built the same model of RT data for the no trials to investigate whether a difference still existed even under the circumstance that that decision strategy could not work. If learners’ decisions were based on word-pair similarity, they would be expected to choose yes when L2 and L1 words were related and no when not, leading to high accuracy on related new meanings in yes trials, whereas as in the no trial each L2 word was paired with a wrong, unrelated L1 word, participants should reject it, no matter which condition it belonged to. Thus, if the RT data for the related meaning condition in the no trials remained significantly different from those for the unrelated meaning condition, it would indicate semantic similarity effects rather than decision strategy effects on word recognition. Our model’s results showed that the no trial data were consistent with those for the yes trial, in which the related meaning condition was recognized faster than the unrelated meaning condition (*b* = 60.322, *t* = 5.717, *p* < 0.001). Thus, the difference we found seems not due to decision strategy either, but to semantic similarity.

However, this study still leaves some questions unresolved. First, the mechanism of the formation of the right mapping relationship from L2 form to unrelated new meaning remains unclear. Although the connectionist model explains why unrelated new meanings were hard to learn, it does not provide a clear explanation of how unrelated new meanings become connected to the familiar L2 form without any shared semantic features. Further studies are needed to investigate the cognitive mechanism of learning unrelated new meanings. Second, the slopes of the growth curve, which we used to reflect the learning, are an indirect way to explore the cognitive mechanism of the learning. More delicate technologies, like ERP and fMRI, are needed to directly investigate the online encoding process during learning. Finally, in the field of ambiguous word learning, the distinction between familiarity and semantic similarity has never been subject to clear focus: whether two meanings are highly correlated due to high co-occurrence frequency in real life or to highly overlapping semantic features is an interesting question that future study can explore.

## Conclusion

Our study explored the impact of semantic similarity on learning of artificially paired new meanings of known L2 words. We found that the slope of learning related new meanings is steeper than that of learning unrelated new meanings, which indicates that the growth speed of learning new meanings is faster when the new meaning is semantically related to the known meaning. The difference in the speeds of learning related meaning and unrelated meaning suggests that semantic similarity mediates meaning learning, which supports the connectionist model. In particular, the shared features between old and new meanings facilitate the procedure of incorporating related new meanings into the lexical semantic network.

## Author Contributions

YZ and BC designed the experiment and wrote the manuscript. YZ and YT collected and performed data analysis. YT, BC, PY, and YL edited and revised the manuscript.

## Conflict of Interest Statement

The authors declare that the research was conducted in the absence of any commercial or financial relationships that could be construed as a potential conflict of interest.

## References

[B1] Armstrong B. C. Plaut D. C. (2008). “Settling dynamics in distributed networks explain task differences in semantic ambiguity effects: computational and behavioral evidence,” in *Proceedings of the Annual Meeting of the Cognitive Science Society*, Austin, TX, 30:30

[B2] Armstrong B. C. Plaut D. C. (2016). Disparate semantic ambiguity effects from semantic processing dynamics rather than qualitative task differences. *Lang. Cogn. Neurosci.* 31 940–966. 10.1080/23273798.2016.1171366

[B3] Atir-Sharon T. Gilboa A. Hazan H. Koilis E. Manevitz L. M. (2015). Decoding the formation of new semantics: MVPA investigation of rapid neocortical plasticity during associative encoding through Fast Mapping. *Neural Plast.* 2015:804385 10.1155/2015/804385 26257961 PMC4519547

[B4] Baayen R. H. Davidson D. J. Bates D. M. (2008). Mixed-effects modeling with crossed random effects for subjects and items. *J. Mem. Lang.* 59 390–412. 10.1016/j.jml.2007.12.005

[B5] Bates D. Maechler M. Bolker B. Walker S. (2014). *lme4: Linear Mixed- Effects Models Using Eigen and S4. R Package Version.* Available at: http://CRAN.R-project.org/package=lme4

[B6] Beijing Language and Culture University (1986). *Modern Chinese Frequency Dictionary.* Beijing: Beijing Language and Culture University Press.

[B7] Bisson M. J. van Heuven W. J. B. Conklin K. Tunney R. J. (2014). The role of repeated exposure to multimodal input in incidental acquisition of foreign language vocabulary. *Lang. Learn.* 64 855–877. 10.1111/lang.12085 25558094 PMC4277705

[B8] Bogaards P. (2001). Lexical units and the learning of foreign language vocabulary. *Stud. Second Lang. Acquis.* 23 321–343. 10.1017/S0272263101003011

[B9] Bracken J. Degani T. Eddington C. M. Tokowicz N. (2017). Translation semantic variability: how semantic relatedness affects learning of translation-ambiguous words. *Bilingualism* 20 783–794. 10.1017/S1366728916000274

[B10] Cunnings I. (2012). An overview of mixed-effects statistical models for second language researchers. *Second Lang. Res.* 28 369–382. 10.1177/0267658312443651

[B11] Degani T. Tokowicz N. (2010). Ambiguous words are harder to learn. *Bilingualism* 13 299–314. 10.1017/S1366728909990411

[B12] Degani T. Tseng A. M. Tokowicz N. (2014). Together or apart? learning of ambiguous words. *Bilingualism* 17 749–765. 10.1017/S1366728913000837

[B13] de Groot A. M. B. (2011). *Language And Cognition In Bilinguals And Multilinguals: An Introduction.* New York, NY: Psychology Press 10.4324/9780203841228

[B14] Gernsbacher M. A. (1984). Resolving 20 years of inconsistent interactions between lexical familiarity and orthography, concreteness, and polysemy. *J. Exp. Psychol. Gen.* 113 256–281. 10.1037/0096-3445.113.2.256 6242753 PMC4311894

[B15] Hothorn T. Bretz F. Westfall P. (2008). Simultaneous inference in general parametric models. *Biom. J.* 50 346–363. 10.1002/bimj.200810425 18481363

[B16] Jager B. Green M. J. Cleland A. A. (2016). Polysemy in the mental lexicon: relatedness and frequency affect representational overlap. *Lang. Cogn. Neurosci.* 31 425–429. 10.1080/23273798.2015.1105986

[B17] Klein D. E. Murphy G. L. (2001). The representation of polysemous words. *J. Mem. Lang.* 45 259–282. 10.1006/jmla.2001.2779

[B18] Klepousniotou E. (2002). The processing of lexical ambiguity: homonymy and polysemy in the mental lexicon. *Brain Lang.* 81 205–223. 10.1006/brln.2001.2518 12081393

[B19] Klepousniotou E. Baum S. R. (2007). Disambiguating the ambiguity advantage effect in word recognition: an advantage for polysemous but not homonymous words. *J. Neurolinguistics* 20 1–24. 10.1016/j.jneuroling.2006.02.001

[B20] Klepousniotou E. Pike G. B. Steinhauer K. Gracco V. (2012). Not all ambiguous words are created equal: an EEG investigation of homonymy and polysemy. *Brain Lang.* 123 11–21. 10.1016/j.bandl.2012.06.007 22819308

[B21] Klepousniotou E. Titone D. Romero C. (2008). Making sense of word senses: the comprehension of polysemy depends on sense overlap. *J. Exp. Psychol. Learn. Mem. Cogn.* 34 1534–1543. 10.1037/a0013012 18980412

[B22] Laufer B. Elder C. Hill K. Congdon P. (2004). Size and strength: do we need both to measure vocabulary knowledge? *Lang. Test.* 21 202–227. 10.1191/0265532204lt277oa

[B23] Liu Y. Dunlap S. Fiez J. Perfetti C. (2007). Evidence for neural accommodation to a writing system following learning. *Hum. Brain Mapp.* 28 1223–1234. 10.1002/hbm.20356 17274024 PMC6871335

[B24] Lu Y. Wu J. Dunlap S. Chen B. (2017). The inhibitory mechanism in learning ambiguous words in a second language. *Front. Psychol.* 8:636. 10.3389/fpsyg.2017.00636 28496423 PMC5406400

[B25] Medler D. A. Binder J. R. (2005). *MCWord: An On-Line Orthographic Database of the English Language.* Available at: http://www.neuro.mcw.edu/mcword/

[B26] Mirman D. (2014). *Growth Curve Analysis and Visualization using R.* Boca Raton, FL: CRC Press.

[B27] Murre J. M. J. (2014). S-shaped learning curves. *Psychon. Bull. Rev.* 21 344–356. 10.3758/s13423-013-0522-0 24065285

[B28] Pu H. Holcomb P. J. Midgley K. J. (2016). Neural changes underlying early stages of l2 vocabulary acquisition. *J. Neurolinguistics* 40 55–65. 10.1016/j.jneuroling.2016.05.002 28983152 PMC5625355

[B29] R Core Team (2013). *R: A Language and Environment for Statistical Computing.* Vienna: R Foundation for Statistical Computing

[B30] Rodd J. M. Berriman R. Landau M. Lee T. Ho C. Gaskell M. G. (2012). Learning new meanings for old words: effects of semantic relatedness. *Mem. Cogn.* 40 1095–1108. 10.3758/s13421-012-0209-1 22614728

[B31] Rodd J. M. Gaskell G. Marslen-Wilson W. (2002). Making sense of semantic ambiguity: semantic competition in lexical access. *J. Mem. Lang.* 46 245–266. 10.1006/jmla.2001.2810

[B32] Rodd J. M. Gaskell M. G. Marslen-Wilson W. D. (2004). Modelling the effects of semantic ambiguity in word recognition. *Cogn. Sci.* 28 89–104.

[B33] Schlichting M. L. Mumford J. A. Preston A. R. (2015). Learning-related representational changesreveal dissociable integration and separation signatures in the hippocampus and prefrontalcortex. *Nat. Commun.* 6:8151. 10.1038/ncomms9151 26303198 PMC4560815

[B34] van Kesteren M. T. R. Ruiter D. J. Fernandez G. Henson R. N. (2012). How schema and novelty augment memory formation. *Trends Neurosci.* 35 211–219. 10.1016/j.tins.2012.02.001 22398180

[B35] Wen Y. van Heuven W. J. (2017). Chinese translation norms for 1,429 English words. *Behav. Res. Methods* 49 1006–1019. 10.3758/s13428-016-0761-x 27325164 PMC5429370

